# Rocks, Dandelions or Steel Springs: Understanding Resilience from a Public Health Perspective

**DOI:** 10.3390/ijerph18158189

**Published:** 2021-08-02

**Authors:** Karl Gauffin, Josephine Jackisch, Ylva B. Almquist

**Affiliations:** Department of Public Health Sciences, Stockholm University, 10691 Stockholm, Sweden; josephine.jackisch@su.se (J.J.); ylva.almquist@su.se (Y.B.A.)

**Keywords:** resilience, childhood adversity, metaphors

## Abstract

The multifaceted concept of resilience is widely used to describe individual or societal abilities to withstand and adjust to external pressures. In relation to health, resilience can help us to understand a positive health development despite adverse circumstances. The authors of this article aimed to disentangle this complex concept by elaborating on three metaphors commonly used to describe resilience. Similarities and differences between resilience as a rock, a dandelion, and a steel spring are discussed. The metaphors partly overlap but still provide slightly different perspectives on the development and manifestation of resilience. With reference to longitudinal studies of long-term health development, the article also elaborates on how resilience relates to temporal dimensions commonly used in epidemiological studies: age, cohort, and period. Moreover, the interaction between resilience at individual, organizational, and societal levels is discussed. In conclusion, it is argued that public health sciences have great potential to further a theoretical discussion that improves our understanding of resilience and promotes the integration of individual- and community-level perspectives on resilience.

## 1. Introduction

The multifaceted concept of resilience appears in a wide spectrum of settings pertinent to both the natural and human sciences. For many researchers in psychology, sociology, and epidemiology, the concept has been defined as a disposition that allows for positive (or the lack of negative) outcomes in circumstances where adverse outcomes would be expected [1,2,3]. The fact that resilience enjoys widespread popularity across academic disciplines can be interpreted as a sign of the theoretical and practical usefulness of the concept, but this cross-disciplinary popularity may also turn resilience into a boundary object characterized by conceptual vagueness [4]. In other words, depending on our academic background, level of analysis, and theoretical or methodological point of departure, we may have different views on the meaning, development, and manifestation of resilience [5,6,7]. Public health research, which is often designed to investigate the effect of social and environmental circumstances on individual and population health, has the potential to make great use of the concept [8,9,10,11]. As a transdisciplinary field of research, public health science has been influenced by a number of neighboring disciplines when analyzing the connection between resilience and health. For example, in studies focusing on how resilience develops in light of childhood adversities, the concept has traditionally been influenced by psychiatric approaches [7]. However, in more recent time, it has also come to encapsulate sociological notions of resilience, highlighting the contextually dependent and modifiable aspects of child development [11].

The authors of this article aimed to further disentangle resilience as used in public health research by elaborating on three commonly used metaphors of the concept. A review of these metaphors and how they can be applied to ongoing public health crises can help to bridge the gap between individual and community applications of resilience, as well as promote understanding of the relationship between resilience and health.

## 2. The Concept of Resilience in Public Health Research: A Theoretical Overview

Theorizing around concepts relevant to health is an activity that engages researchers from a large number of disciplines. The intellectual exercise of developing and reflecting on theory, or theorizing, is a disciplined activity that requires a combination of imagination and a sense for practical congruity, as well as an appreciation of both the abstract and concrete [12]. In the pursuit of useful theory, an individual researcher may run into a number of difficulties: the task at hand may seem opaque and the question of what should actually be done may be hard to answer. As theoretical accounts—with notable exceptions—tend to focus on the outcome (theory) rather than the process (theorizing), this widespread confusion is unsurprising [13]. Nonetheless, theory development within the public health sciences is a worthwhile endeavor: a defined theoretical framework provides researchers with a paradigmatic point of reference, and theory also serves as a lens through which we may interpret our empirical results and as a means of generating new knowledge [14]. It is worth noting that comprehensive theorizing around resilience is a massive task that is beyond the scope of a single paper. Rather, this article aims to comment on the use of resilience in public health research and by doing that, contribute to a platform for further theoretical discussion.

In general, resilience as applied to humans and societies is always fundamentally a metaphor, reminding us of the origin of the concept in the natural sciences to describe the ability of a material or system to absorb or avoid damage (from the Latin *resilire*; to spring back or rebound) [15]. The growing interest in resilience in public health research has resulted in a number of literature reviews defining the way in which resilience connects to health and wellbeing within specific fields, e.g., for individuals [16] or communities [17], and how it relates to post-traumatic growth [18], child abuse [19], and the operationalization of resilience in longitudinal studies [20]. In public health research, the conceptualizations of resilience can broadly be divided into two major streams of literature. The first focuses on the individual’s capacity of bouncing-back from stress and adversity [16,21], and the other is concerned with the resilience of systems (e.g., disaster and community resilience), which, in public health research, is related to emergency preparedness and features of communities to resist and recover from human or natural hazards and crises [15,17,22]. Building on these reviews, we posit here that public health sciences may be exceptionally well placed to embrace the conceptual complexity of resilience and combine its individual- and community-level perspectives. Such an integrated view would position biological and psychological factors in the social and wider societal context, which together determine the capacity to adapt in the face of adversity or change [23].

### 2.1. An Iconography of Resilience

One way to shed further light on resilience in public health research is to turn to the use of more specific visualizations to narrow the general resilience metaphor. Iconographies and metaphors can prove useful to illustrate central constructs of a concept, as well as to give information on how to appropriately apply the concept in empirical research and practical work [24,25]. The metaphors can help us make distinctions regarding the development and manifestation of resilience. The development of resilience touches on questions around the origin and explanatory power of the concept: why does resilience emerge and where does it come from? The manifestation of resilience, on the other hand, surrounds the question of realization: when and how does resilience show itself? This second question is of high relevance for the model development and operationalization of resilience in public health research. The literature mentions three partly overlapping constructs of resilience in public health research, which can be illustrated by three metaphors: the rock, the dandelion, and the steel spring.

Resilience as a rock encapsulates a traditional conception of resilience that focuses on attributes such as sturdiness or robustness [1,26]. A rock-resilient individual is a strong individual who will remain relatively unaffected by any adverse exposure. Unlike other notions of resilience, the rock metaphor invites to the idea of a fairly static type of toughness that will serve as a protective factor throughout time and in all circumstances. As such, the rock metaphor does not pick up the idea of resilience being dependent on context. Rather, it exists independently from any challenges, and if applied to an individual, rock resilience can be understood as a sort of personality trait that has developed detached from any outer circumstances. In empirical studies, it can be operationalized as a moderator with a protective potential across different contexts and situations. Later literature has widely dismissed this conception of resilience, as it lacks any consideration of the flexibility that is central to other resilience constructs [26]. Rock resilience is also immune to any policy efforts: either an individual is or is not born resilient; this is nothing that external factors can promote. It can also be argued that the understanding of resilience along the lines of the rock metaphor may be ethically questionable, since it implies a notion of bulletproof invulnerability, which ignores the suffering that resilient individuals go through in times of hardship. Despite these problematic aspects of the metaphor, resilience as a rock remains as an idea in some public health-related research when referring to individuals who seem unshaken by any types of experienced adversities.

The second commonly used metaphor is that of the dandelion. This idea of resilience is commonly used to describe an individual, often a child, who develops in a positive way despite difficult surroundings [27]. Just like a flower growing in the cracks of the pavement, a dandelion-resilient person has the ability to flourish even if their environment is harsh. Comparable to the rock metaphor, certain pre-existing traits enhance the capacity to adapt to environmental stress in the dandelion, but this may be dependent on context, available resources, and situation. Dandelion resilience at the individual level is close to what has been called ‘emergent resilience’ in the literature, and adversities in the context of the dandelion typically refer to rather chronically adverse conditions [28]. Dandelion resilience can be operationalized as a factor that positively modifies the effect of an adversity on an outcome, whereby its potential as a mitigating buffer depends on the contextual setting—unlike rock resilience, which provides a static and unalterable protection. Sometimes, the dandelion is accompanied by other flowers in metaphorical attempts to describe personality traits and children’s vulnerability. While the dandelion child is regarded to be the resilient type, the orchid child is distinguished by high sensitivity and the need for optimal circumstances to flourish. In some accounts, the tulip has been used to describe a personality type in between the resilient dandelion and the sensitive orchid [27,29]. At this point, it could also be worth noting that the flower typology of children may be interconnected to ideas and presumptions regarding child behavior and social class. The orchid metaphor describes a troubled but inherently refined person, and it may therefore, in everyday discourse, more often be used to signify developmental difficulties, such as learning disabilities, in more privileged children. The same problems in working class children, on the other hand, may be less likely to become euphemized in this way.

Finally, the metaphor of the steel spring invites us to make a stronger connection between resilience and recovery. In contrast to the idea of resilience as an inherent feature of individuals, we can understand it as a dynamic process or as a reaction to external events. In this conception of resilience, the adversity (e.g., a life crisis) makes the individual develop qualities that they would not have developed in the absence of adversity. Only under pressure, the individual—just like the steel spring—will activate its resilience, maintain, and eventually bounce back to a state of homeostasis after temporal perturbation in functioning. Naturally, these abilities are often found in individuals or organizational units that would also be defined as resilient in the dandelion meaning. Thus, the conceptualizations are overlapping rather than contradictory, but they draw our attention to slightly different aspects of resilience. For example, while the dandelion metaphor points to the ability to growth or development despite adverse circumstances, which in turn results in a consolidated adaptation to the environment, the idea of steel spring resilience focuses on abilities for recovery or re-organization after the experience of collapse or substantial stress. The steel spring metaphor is most readily used for disasters or other shocks to a system, such as in adults who are confronted with a (often sudden) particular stressful life event like experiences of war, assault, fatal sickness, or loss of a loved one. As such, it can be compared with the so-called ‘minimal impact resilience’, which is used to describe a disposition that prevents a lasting negative effect of singular acute life events [28]. Methodologically and empirically, steel spring resilience can only be determined after exposure to stress, adversity, or disaster. It could be operationalized in terms of both the process and a positive outcome (or the lack of an anticipated negative outcome), meaning that health is regained despite significant stress and adversity. The steel spring metaphor is limited in the sense that it seems to only allow for a return to the same state as before. Resilience could, however, also be expanded to include something that has been called post-traumatic growth, namely the possibility of emerging stronger after exposure (like if a steel spring could improve capacity after it had been employed). Though theoretically and culturally popular, the idea of post-traumatic growth has been contested in empirical accounts [18], indicating that the timing and conditions of the adaptation process become relevant research questions to investigate. The public health implications of using the steel spring resilience concept would be to focus on promoting supportive environments, which can be rich in resources and provide general environmental conditions that foster positive adaptation, as well as providing access to individual support through structures that specialize on recovery.

### 2.2. Resilience over Time and Space

Given the many different parameters that may be considered, engaging in the concept of resilience from a public health perspective is a fruitful exercise. Based on the various definitions of resilience, it is generally acknowledged that our understanding of resilience may depend on our units of analysis and temporal dimensions [2]. At least three units of analysis come to mind: individual, organizational, and societal. Individual resilience usually refers to a person’s ability to cope with the environment they find themselves in, organizational resilience may refer to an administrative entity (e.g., a health care system), and societal resilience can point to the collective ability of communities to withstand the pressure of external crises [30]. Table 1 summarizes the ways the three metaphors of resilience may be manifested on individual, organizational, and societal levels.

Paying increased attention to the interactions between individuals and their environments may also shed further light on the difficult question regarding the origin of resilience. A challenge for researchers in multidisciplinary fields, such as public health sciences, is the need for expertise in several, often quite diverse, academic areas. As this high level of combined knowledge is rarely achieved by an individual researcher, or even by research groups, the complex interactions between biology and environment tend to become oversimplified. Whereas a public health researcher with a background in genetics, medicine, or clinical psychology may reduce all social, environmental, and ecological determinants to a common pool of external confounders that distorts the relationship between genes, physiology, or human psychology and health, a background in social sciences may lead to the very same simplifications but with regard to the complexities of human biology.

In terms of temporal dimensions, we may consider the classical time-varying elements in epidemiological studies: age, period, and cohort. Age effects are usually understood as variations connected to the biological and social process of aging, which are specific to individuals. In studies of age and resilience, we may therefore hypothesize that resilience is developed and manifested at different stages of a person’s life. We could, however, also think about whether the resilience of an organizational or structural unit of analysis is a function of how long that organization has existed. Here, a more mature or well-established organization could potentially be seen as more resilient than a new one. Period effects are commonly defined as external factors affecting all units of analysis, independent of their age or maturity. Wars, economic crises, or pandemics may be examples of events with potential impact on the development and manifestation of resilience in individuals, organizations, and societies. Cohort effects can be described as the interactions between age and period effects, where certain age groups may be more affected by specific external periodic events. Investigating resilience in relation to dimensions of time and different units of analysis adds complexity to a multifaceted concept, but the usefulness of epidemiological terminology also demonstrates that resilience and public health research are a good match [31]. In public health research, some of the most widely used conceptual models—such as the Dahlgren and Whitehead rainbow model [32], Bronfenbrenner’s ecological systems theory [33], Krieger’s ecosocial theory [34], and the life course cube introduced by Bernadi et al. [35]—were developed to illustrate how multiple levels across time and space interact to impact population health. Revisiting these classical models may be helpful in order to give further meaning to the resilience concept.

### 2.3. Resilient Individuals and Resilient Societies in a Changing World

As has become painfully clear in the ongoing climate crisis, health and wellbeing are highly dependent on changes in the ecological environment. In fact, contemporary perspectives on resilience regard individuals and their social environments to be completely embedded within the ecological environment [36]. Going further than conventional approaches in classifying the ecological environment as one of many factors contributing to human wellbeing, implementing a social–ecological approach to resilience means acknowledging that human health and wellbeing rest on the capacity of the biosphere to sustain us, as well as on our ability to adapt or transform in face of ongoing, and often unexpected, change in social–ecological systems [37]. For multiple reasons, the climate crisis is a potential aggravator of social inequalities [38], in which the interplay between individual, organizational, and societal resilience will be of central importance. This impending, uncertain future calls for reflection on how the interplay between resilient individuals and societies will look. Again, we can make use of the metaphors to illustrate different manifestations of resilient societies.

A rock society would remain invulnerable and statically robust, no matter the nature and severity of the adversity it is facing. Though no such society really exists, it has for long signified the conventional approach to societies and, perhaps even more so, to the ecological environment. In light of the climate crisis, it has become more and more evident that this approach to resilience has become obsolete. A dandelion society, in contrast, would be characterized not by invulnerability but by flexibility and the ability to adapt to chronic adversity. The dandelion’s capacity to thrive under meagre circumstances could be translated into the efficient societal use of natural and other types of resources. Reflecting on dandelion resilience in the individual, this type of resilience at the structural level could be seen as a pre-existing quality making a society well-equipped to deal with external pressures and crises. Finally, a steel spring society will demonstrate its abilities in the time immediately following a crisis, as the main focus here is the ability to recover or bounce back after times of acute adversity. Again, the metaphors of the dandelion and the steel spring should not be seen as mutually exclusive. In fact, speedy recovery should preferably be combined with adaptability, meaning, in the case of societies, not going back to business as usual but adjusting to a new post-crisis reality and preparing for the future.

The COVID-19 pandemic is another public health crisis that clearly illustrates the interdependencies between individual, organizational, and societal resilience. Policies were designed to promote resilience on all three levels. Individuals were given preventative tools and information to handle the adverse situation of a rapidly spreading infectious disease. These individual prevention efforts helped health care systems to continue operating during a prolonged period of medical emergency. At the same time, changes in care facilities’ procedures and protocols have increased organizational resilience. The efforts from both individuals and organizations—in conjunction with broader political measures and stimulus packages—thereby permitted societal resilience, i.e., have allowed basic public health, economic, and social structures to function during the pandemic. Similarly, a resilient society increases the capacity of organizations and individuals to remain resilient and continue to function. The three metaphors can be used to make further distinctions between the types of resilience in the pandemic response. Whereas rock resilience may take the perseverance of individuals, organizations, and societies throughout the COVID-19 crisis for granted, dandelion resilience may promote flexible adaptation to the pandemic. Conversely, steel spring resilience may signify the hope that individuals, organizations, and societies can quickly bounce back to how things were before. On the other hand, in light of the climate crisis, a return to ‘business as usual’ is, for many reasons, not desirable. It remains to be seen which metaphor of resilience best encapsulates the need for adaption, recovery, and the type of sustainable change, which is indispensable for a livable future.

## 3. Conclusions

The authors of this article investigated the complex concept of resilience from a public health perspective, positing that public health sciences should embrace this complexity by integrating individual- and community-level perspectives of resilience while refining conceptual clarity. Following the tradition of iconographies and metaphor use in public health research, three types of resilience—the rock, the dandelion, and the steel spring—were presented. In an attempt to integrate the individual and community perspectives, we also discussed the concept in sociological and epidemiological terms as it relates to different units of analysis (individual, organizational, and societal) and temporal dimensions (age, period, and cohort). The importance of resilience at the individual, organizational, and societal levels will be of continued importance in the turbulent times ahead. This article underlines both the relevance of public health sciences for understanding resilience and the relevance of resilience for the advancement of public health research in the future.

## Figures and Tables

**Table 1:** The metaphors of resilience in individuals, organizations and societies.

	Rock	Dandelion	Steel Spring
**Individual**	Invincibility.	Adaptability and compensatory potential.	Ability to recover.
**Organizational**	Unaffected by any external pressure.	Focus on emergency preparedness.	Ability to quickly regain operational capacity following a crisis.
**Societal**	Statically robust.	Focus on security, risk minimization, and disaster preparedness.	Focus on recovery, mitigation, and appropriate counter measures.

## Data Availability

Not applicable.
